# Safe Service Delivery of a Complex Early Pregnancy Problem: Caesarean Scar Pregnancy

**DOI:** 10.3390/jcm11237063

**Published:** 2022-11-29

**Authors:** Hanine Fourie, Ahmad El-Zibdeh, Victoria Heppell, Ingrid Granne, Lee Nai Lim, Prasanna Raj Supramaniam

**Affiliations:** 1 John Radcliffe Hospital, Oxford University Hospitals NHS Foundation Trust, Headley Way, Oxford OX3 9DU, UK; 2 Early Pregnancy Assessment Unit, Oxford University Hospitals NHS Foundation Trust, Oxford OX3 9DU, UK; 3 Nuffield Department of Women’s and Reproductive Health, Oxford University, Oxford OX1 2JD, UK

**Keywords:** caesarean scar pregnancy, ultrasound, early pregnancy loss, service provision

## Abstract

Caesarean Scar Pregnancy (CSP) is an ectopic pregnancy with implantation into the niche of the uterine scar. We aimed to describe the local management of consecutive cases of CSP to develop a standard operating procedure (SOP). Between December 2019 and June 2022, there were 19,100 maternities. Of these, 23 were CSPs in 19 patients. Median BMI was 29 (range 20.5–52), median number of Caesarean deliveries (CS) was 2 (range 1–4) and 7/23 (30%) were cigarette smokers. At diagnosis, 9/23 were live pregnancies, 3/23 were retained products of conception (RPOC), 9/23 were pregnancies of uncertain viability (PUV), and 2/23 were non-viable. In six, the initial management was expectant, surgical suction evacuation with transrectal ultrasound guidance in 16, and one had a hysterectomy. The median blood loss was 100 mL (range 50–2000 mL). Two patients (9%) required a blood transfusion. Median hospital stay was 1 day (range 0–4). At follow-up after 10 weeks, no patients had an ongoing haematoma, and one had significant RPOC electing hysterectomy. Eight women were known to have 9 subsequent pregnancies (recurrent CSP *n* = 4, livebirth *n* = 2, miscarriage *n* = 2, tubal ectopic *n* = 1). Outcomes as rated by low blood loss, short hospital stay, and rare need for further intervention were favorable. Factors associated included prompt ultrasonographic diagnosis, availability of transrectal ultrasound guided surgery, and specialist follow-up, which form the basis of the SOP.

## 1. Introduction

Rates of caesarean (CS) deliveries have increased globally [1] and therefore risks of CS in subsequent pregnancy have received increasing attention [2]. Multiple caesarean deliveries confer additional risk in subsequent pregnancies with one study showing a 0.19% risk of placenta accreta after one CS, increasing to 9% after four or more [3].

Caesarean Scar Pregnancy (CSP) is an ectopic pregnancy [4] with anterior implantation into the niche of the uterine scar. As per recent Delphi consensus, CSP can be classified as Type 1 where the largest part of the gestational sac (GS) protrudes towards the uterine cavity; Type 2 where the largest part of the GS is within the myometrium not breaching the serosa, and Type 3 where the pregnancy is partially located outside the contour of the cervix or uterus [5].

Abnormal implantation in ongoing pregnancy, miscarriage, or in retained products of conception, is increasingly recognized with wider availability of early pregnancy care [6]. However, accurate diagnosis, management, and follow up relies on a specialist multidisciplinary team (MDT) with access to high quality ultrasonography.

We conducted a prospective cohort study in our tertiary center with the aim of developing a standard operating procedure, which could have utility more broadly.

## 2. Materials and Methods

In 23 consecutive cases of CSP between December 2019 and June 2022, we reviewed patient factors, diagnosis, and management as a prospective cohort study. This background was used to propose a SOP.

**Figure 1:**
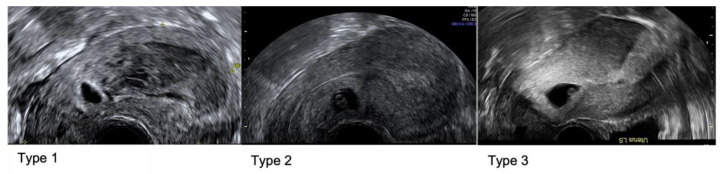
Caesarean Scar Pregnancy types as per Delphi consensus: Type 1 where the largest part of the gestational sac (GS) protrudes towards the uterine cavity; Type 2 where the largest part of the GS is within the myometrium but not breaching the serosa, and Type 3 where the pregnancy is partially located outside of the contour of the cervix or uterus.

Initial ultrasound assessments were made by sonographers in a community early pregnancy unit. Low implantation with proximity to the CS scar and an empty uterine cavity and endocervical canal, prompted discussion at the local early pregnancy MDT. Subsequent assessment was then performed by a specialist gynaecology consultant trained in advanced early pregnancy and gynaecology ultrasound. During the specialist ultrasound assessment, the mean size of the GS and CRL were documented if present, and the presence of a yolk sac and/or amniotic sac were noted. In RPOC, the mean size was documented. The colour Doppler flow was described with a score between 1–4 [7] with 1 representing no flow around the pregnancy, 2 minimal flow, 3 moderate flow and 4 significant vascularity. The residual myometrial thickness (RMT) was measured at the level of implantation. The degree of invasion was also described in relation to the uterine serosa. Retrospectively the cases were described as Type 1, 2 or 3 as per recent Delphi consensus (Figure 1) [5].

Patients with CSP were counselled by a member of the specialist team consisting of three gynaecology consultants. They were offered expectant or surgical management. As per RCOG guidance, sole medical management with intramuscular methotrexate was not offered [4]. Expectant management was offered with either the expectation of spontaneous pregnancy loss or the expectation of abnormally invasive placenta in live pregnancy with referral to a specialist fetal medicine placenta clinic [8].

Surgical management was performed by a team of two consultant gynecologists and a consultant anaesthetist, with methodology inspired by Jurkovic et al.[9] The procedure was performed under general anaesthesia with appropriate surgical prophylaxis, in accordance with local antimicrobial trust guidelines. Initially, a “McDonald” cervical cerclage using a Prolene-1 suture was inserted. Then, under transrectal ultrasound guidance, cervical dilatation using a Hegar’s dilatator and suction evacuation using a rigid cannula was performed. After removal of the tissue with suction evacuation, the caesarean scar niche and the uterine cavity was carefully inspected to ensure complete removal. There was often a small amount of active bleeding in this area which provided a sonolucent window illustrating the endometrial and endocervical lining clearly. The cervical suture was then clipped temporarily to assess for active bleeding. The CSP area was reviewed using transrectal ultrasound scan after 15 min. The cervical suture was removed if there was no bleeding. In cases of a non-expanding haematoma and no ongoing active bleeding the suture was tied. If there was evidence of ongoing bleeding, a Foley’s catheter was inserted, inflated and used for balloon tamponade against the CSP scar. The Foley catheter was routinely removed the following day. In women where the cerclage was tied, the cerclage was removed 3 days later with an ultrasound assessment. In patients where RPOCs were identified following this surgical approach; options including expectant, medical and surgical management of RPOCs were offered.

Follow up plans were individualized. All patients were invited for follow up 6 weeks later which included an ultrasound assessment. Serum βHCG quantification was only used to aid diagnosis when RPOC was suspected.

## 3. Results

**Figure 2:**
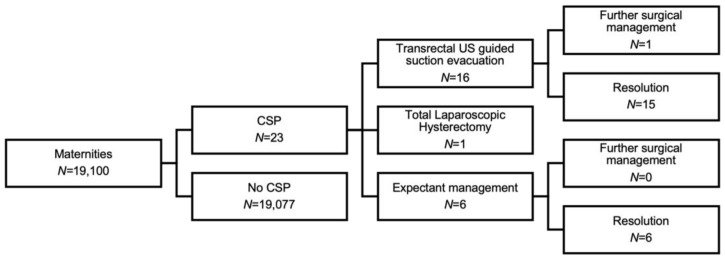
Flowchart of Caesarean Scar Pregnancy cases and management.

In our unit, the estimated incidence of CSP was 1.2 in 1000 (23/19,1000) maternities. Figure 2 illustrates the CSP cases and subsequent clinical management.

### 3.1. Demographic Features

The median age of patients was 34 years (range 28–43). On average, patients were overweight with a median body mass index (BMI) of 29 (20.5–54). Seven women reported cigarette smoking (30.4%). Five women reported a medical condition (depression *n* = 3, hypertension *n* = 1 and antiphospholipid syndrome *n* = 1). As well as previous caesarean, six women reported other gynaecological surgery (surgical management of miscarriage *n* = 3, surgical management of CSP *n* = 1, large loop excision of transformation zone *n* = 1 and marsupialisation of Bartholin’s gland *n* = 1). By definition patients were multiparous with median gravidity of 4 (range 2–9) and median parity of 2 (range 1–4). Eleven patients (57%) had experienced pregnancy loss prior to CSP. The median number of CS prior to studied pregnancy was 2 (range 1–4). The minority of patients (7/19, 37%) had one CS prior to CSP, and of these 3/7 were at a preterm gestation (indication vasa praevia *n* = 1, intrauterine growth restriction *n* = 1, severe COVID-19 infection *n* = 1). Of the patients with 2 or more CS, none were performed at a preterm gestation.

### 3.2. Symptoms Leading to Ultrasound Assessment

Fourteen patients (61%) experienced bleeding and pain in early pregnancy. Five (22%) experienced bleeding only, with two presenting with persistent bleeding following termination of pregnancy. The other four had early pregnancy ultrasound assessment for reassurance (following previous CSP *n* = 2, significant pelvic adhesions *n* = 1, anxiety *n* = 1).

### 3.3. Diagnosis

The median number of ultrasound assessments to make a diagnosis was 1 [1,2,3,4,5]. Certain menstrual dates were available for 20/23, 2 were unable to recall their last menstrual period (LMP), and one conceived on the combined oral contraceptive pill. Of those with certain LMP, the median gestational age was 48.5 days (range 17–82). At the time of diagnosis, nine were characterized as pregnancy of uncertain viability (PUV), nine were live pregnancies, three were RPOC and two were miscarriages. At the time of a specialist consultant scan, the ultrasound features of the pregnancies were documented as per Table 1.

### 3.4. Counselling

Counselling was standardised and provided by one of three gynaecologists. In live pregnancies, options were expectant or surgical. Women with ongoing CSP were made aware of the almost certain antenatal diagnosis of placenta acreta or percreta and the counselling was based on the RCOG Green-Top Guideline [10]. In brief, they were informed of the risk of major haemorrhage, death, preterm birth, caesarean hysterectomy or subsequent hysterectomy (in >50% of cases). It was stressed that ongoing pregnancy requires surveillance in a tertiary unit with access to a MDT including specialists in placenta accreta spectrum disorder and a neonatal intensive care unit. Although surgical management carries the complexity of pregnancy loss, the risk of major haemorrhage and hysterectomy is lower compared to expectant management [9,11,12]. As with live pregnancy, in pregnancies characterised as PUV or RPOC, expectant and surgical management were also offered. Patients who were expectantly managed with anticipation of spontaneous resolution were invited for follow up 2–4 weeks after diagnosis. With awareness that these are difficult decisions, patients were given at least 24 h to reflect on their options before finalising a plan to ensure informed consent.

### 3.5. Management

The management is summarised in Table 2. Of the six patients who opted for expectant management, ultrasound characterisation was PUV (*n* = 5) or RPOC (*n* = 1) where spontaneous resolution was anticipated. Four did not attend for follow up ultrasound assessment but reported cessation of bleeding and negative urinary pregnancy tests. Of the two who had subsequent ultrasound assessment, this was performed two weeks after diagnosis, and in both complete resolution of the CSP was confirmed. One additional patient (not included in this series due to ongoing mid-trimester pregnancy prior to submission) was diagnosed with a live CSP at 9 + 2 weeks and elected expectant management under fetal medicine surveillance.

**Figure 3:**
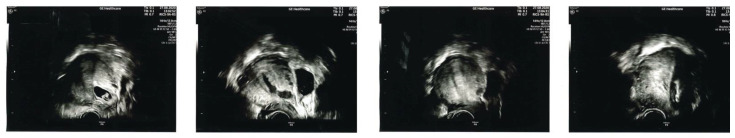
Intraoperative transrectal ultrasound guidance is crucial to ensure complete evacuation of the uterine cavity and caesarean scar niche.

Seventeen patients elected to have surgical management. Fifteen opted for transrectal ultrasound guided suction evacuation (Figure 3). 5/15 required cerclage and balloon tamponade and three required cerclage tamponade only.

**Figure 4:**
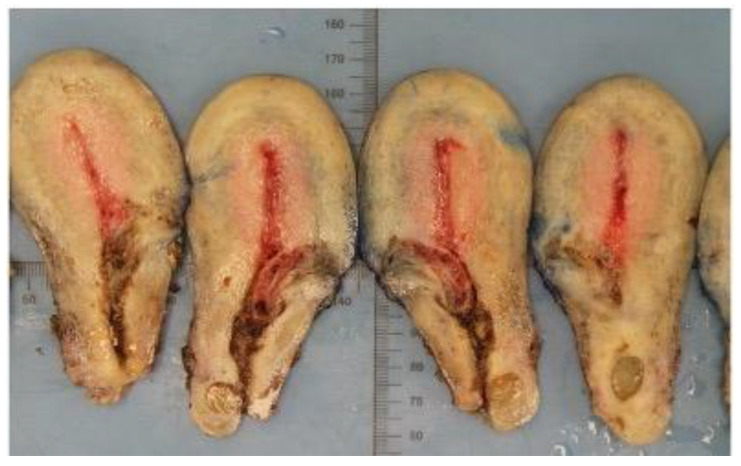
Histopathological hysterectomy specimen where invasive retained products of conception was present following medical termination of pregnancy. Photograph by Dr. Danah Saif.

One had a surgical termination of pregnancy and diagnosis of CSP was made during management of a secondary haemorrhage. The RPOC was managed conservatively. One patient elected to have a total laparoscopic hysterectomy at diagnosis of CSP. This was not a routine management option. In her case, her family was complete, and she had significant RPOC following a medical termination of pregnancy (MTOP). There was sonographic evidence of trophoblast breaching the uterine serosa with invasion of the broad ligament 8 weeks after her initial procedure. Her serum βHCG was raised at 13,559 IU/mL. Although she was offered suction evacuation as a fertility sparing management option, she chose hysterectomy to avoid the ongoing risks of persistent RPOC, bleeding and need for further surgery. The procedure was uncomplicated with a blood loss of 500 mL. Figure 4 shows a hysterectomy specimen following management of CSP.

Overall, median blood loss when recorded was 100 mL (range 50–2000 mL). Median hospital stay was 1 day (range 0–4). Two patients required a blood transfusion.

### 3.6. Follow Up

All women were invited for a follow up appointment 6 weeks after management on average. Sixteen attended follow up. The five patients who had surgical management with cervical cerclage, also attended for follow up on day 3 when an ultrasound scan was performed and the cerclage was removed. Histopathological confirmation of products of conception with no gestational trophoblastic disease was confirmed in all 15 cases who had suction evacuation. In the patient who had a TLH, histopathology showed evidence of a CSP with focal serosal perforation and no evidence of gestational trophoblastic disease.

**Figure 5:**
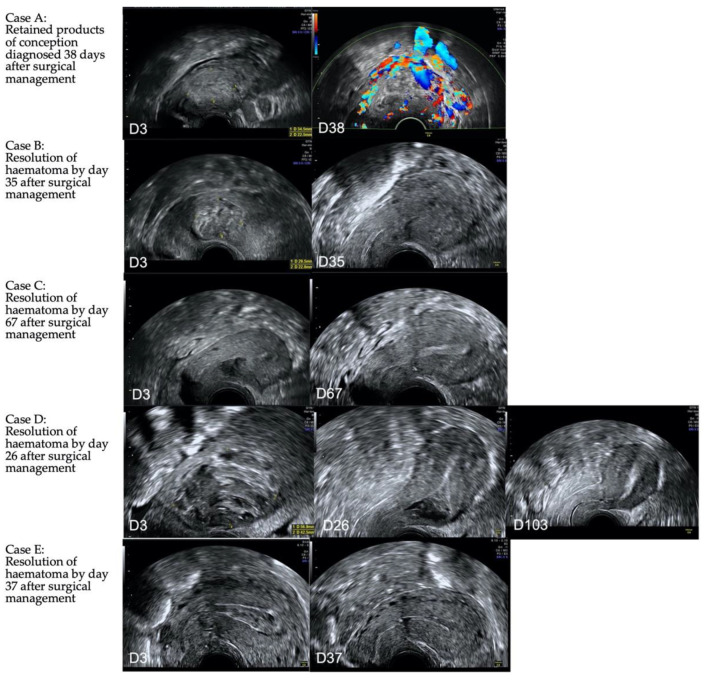
Longitudinal ultrasound images of five cases (case **A**–**E**) managed surgically with cerclage and balloon. The initial image was obtained on day 3 (D3) at the time of cerclage removal, and subsequent images are obtained between day 26 and 103 post surgical management. In 4/5 the haematoma present on day 3 resolves, whereas in one (**A**), retained products of conception is diagnosed at day 38 post surgery.

Following surgical management, formation of an intrauterine haematoma was expected, and in the majority of cases this resolved by the 6-week follow up. In one patient, RPOC was present (Figure 5).

**Figure 6:**
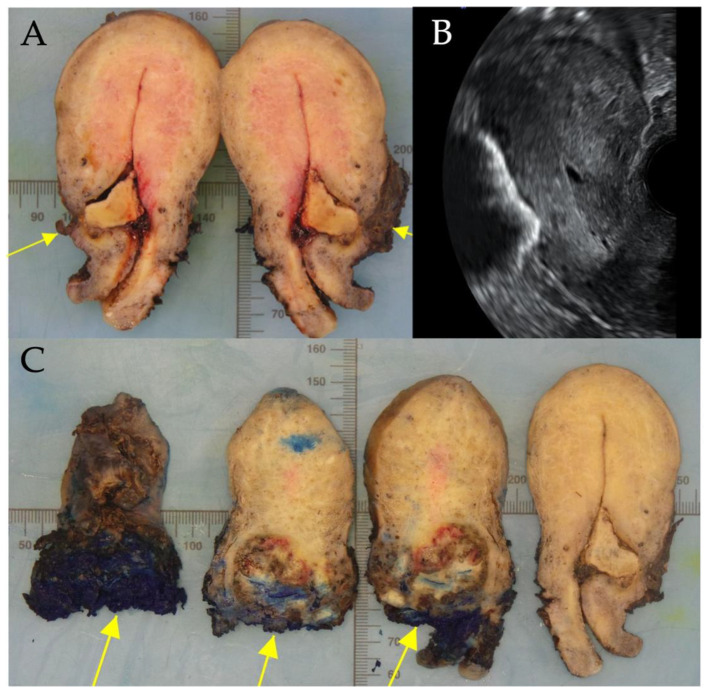
(**A**) Vertically transected hysterectomy specimen with trophoblast in the uterine niche measuring 20 × 15 mm^2^. (**B**) B-mode sagittal view of the uterus correlating with (**A**). (**C**) Hysterectomy specimen with a blue inked appearance illustrating perforation of the left uterine isthmus by trophoblast measuring 56 × 44 mm^2^. Photograph by Dr. Maria Rosario Oliviera Diz.

In the patient with retained products of conception at follow up, the initial management was suction evacuation with cerclage tamponade. As expected, a haematoma was visualised on ultrasound on day 3 post surgery. Five weeks later this area appeared expanded and vascular with a colour score of 4 measuring 31 mm in mean diameter. The serosa was intact. A urinary pregnancy test was positive and βHCG quantified as 2657 U/mL. She was offered expectant, medical and repeat surgical management with suction evacuation. She elected expectant management and had weekly follow up. Eight weeks following surgery, she developed acute pelvic pain. On ultrasound the serosa appeared thin with expansion of trophoblast to the left uterine artery. Therefore she was offered uterine artery embolisation and hysterectomy. Histology showed trophoblast perforating the left uterine wall at the isthmus but no gestational trophoblastic disease. Figure 6 shows a hysterectomy specimen with retained products of conception following management of CSP.

### 3.7. Future Pregnancy

Four women were known not to seek future pregnancy (long term contraception *n* = 2, hysterectomy *n* = 2). Eight women were known to have 9 subsequent pregnancies (recurrent CSP *n* = 4, livebirth *n* = 2, miscarriage *n* = 2, tubal ectopic *n* = 1).

## 4. Discussion

We propose the SOP as outlined below for the management of this rare condition. This SOP is based on our review of consecutive cases over a 30 month period at a tertiary center. It is further referenced by expert consensus and discussion, drawing on the work of Jurkovic et al. [9], data from the national cohort study in the UK[12] and the proposed Tommy’s National Miscarriage Care Package.

1. Patients in the first trimester of pregnancy with a history of CS and symptoms (bleeding ± pain) or a previous CSP, should be offered a transvaginal ultrasound scan from 6 weeks of pregnancy.
2. If there is concern about low implantation close to the CS scar, the case should be reviewed in an early pregnancy multidisciplinary meeting led by a gynaecologist specialising in early pregnancy ultrasound. If suspicion is that of a CSP, the patient should have a detailed ultrasound assessment by a specialist gynaecologist.
3. If the diagnosis of CSP is confirmed, the patient should be counselled about her options and given time to consider her wishes. The patient should have access to the clinical team following initial consultation.
4. If surgical management is performed, intraoperative transrectal ultrasound guidance is crucial to reduce the risk of retained tissue, bleeding and uterine perforation. Post operative intrauterine haematoma is common and can be difficult to differentiate from retained products of conception. Therefore, objective sonographic evidence of complete uterine evacuation at the time of the procedure improves postoperative investigation and diagnosis.
5. Following surgical management, patients should have access to the clinical team due to the risk of ongoing bleeding and infection. A follow-up transvaginal ultrasound should be performed after 6 weeks, with anticipation of resolution of an intrauterine haematoma. At this assessment, the myometrial defect from CS should be assessed. In cases of recurrent CSP and a large myometrial defect, surgical repair of the defect can be discussed.
6. If a patient elects for expectant management of a live CSP, antenatal care should be provided in a tertiary centre with expectation of delivery by complex surgery at a preterm gestational age.
7. All women should be offered psychological support following their pregnancy loss. Patients are given the email address of a specialist early pregnancy counsellor and directed to pregnancy loss charities.
8. All women should be offered contraception if they do not have a desire for future pregnancy, or wish to delay future pregnancy.
9. Women should be counselled about lifestyle interventions to reduce the risk to future pregnancy. Themes include folic acid use, diet and exercise with an aim of normalising BMI, and smoking cessation. Smoking is specifically harmful to pregnancy and is known to be associated with miscarriage[13], preterm birth[14], CSP [15], and other placental disorders of pregnancy[16] which underlies widespread international and national recommendation to reduce maternal smoking [17].

## 5. Conclusions

Collaborative efforts such as the CSPregistry [18] and The UK Early Pregnancy Surveillance Service (UKEPPS) [12] have been important developments to determine evidence-based diagnostic criteria and treatments on larger cohorts of this rare condition. However local management is shaped by available resources and therefore outcomes may not be directly comparable between units. Here, we describe our recent experience of CSP management to address our local need for a SOP. We demonstrate the importance of a multidisciplinary team in providing a safe patient-centred service, which can be adopted in other units. Addressing lifestyle factors such as weight, smoking and birth spacing may reduce the incidence. However, once the placenta invades in or over the caesarean scar niche, early diagnosis with ultrasound, and prompt management in the first trimester, can limit the associated morbidity.

## Figures and Tables

**Table 1:** Ultrasound characteristics of cases at time of specialist ultrasound assessment.

Case	Type of Pregnancy	Mean Sac Diameter (mm) or RPOC Mean (mm)	Crown Rump Length (mm)	Residual Myometrial Thickness (mm)	Colour Score (1–4)
1	PUV	10	NA	UK	UK
2	PUV	33.1	2.5	5.4	2
3	Live	18.8	6.7	3.6	4
4	Miscarriage	-	24.7	3.2	3
5	Live	23.9	14.6	2.8	3
6	RPOC (after PUV)	18	NA	1.9	2
7	PUV	10.05	NA	2.3	2
8	Live	14	4.99	2.5	2
9	RPOC (after PUV)	7.7	NA	9.5	2
10	Live	10..7	3.56	UK	2
11	PUV	15.6	NA	3.6	2
12	Live	17.5	3.08	2.9	4
13	PUV	12.6	NA	3.3	4
14	PUV	22.8	NA	2.6	4
15	Live	18.4	21.46	4.3	2
16	RPOC	9.7	NA	UK	2
17	Live	8.4	2.7	2.7	4
18	RPOC	49.7	NA	2.0	1
19	PUV	5.2	NA	UK	UK
20	Live	12.9	4.7	2.7	4
21	Live	14	UK	5.3	4
22	RPOC	28.8	NA	1.6	4
23	Miscarriage	25.3	17.6	7.3	2

PUV = Pregnancy of Uncertain Viability. RPOC = retained products of conception. NA = not applicable. UK = unknown.

**Table 2:** Management of the 23 Caesarean Scar Pregnancy cases.

Case	GA at Diagnosis (Days)	Type of Pregnancy	Type of CSP	Management	Blood Loss	Complication	Future Pregnancy
1	38	PUV	2	Expectant	NA		Yes
2	33	PUV	2	Surgical	200		Yes
3	37	Live	1	Surgical	600		Yes
4	48	Miscarriage	1	Surgical	2000	Blood transfusion	NA
5	54	Live	2	Surgical	300		NA
6	43	PUV	1	Expectant	NA		NA
7	39	PUV	2	Expectant	NA	Blood transfusion	Yes
8	53	Live	2	Surgical	50		Yes
9	51	PUV	1	Expectant	NA		Yes
10	NA	Live	2	Surgical	50		NA
11	77	PUV	2	Surgical	50		NA
12	41	Live	3	Surgical	100	RPOC requiring hysterectomy	NA
13	NA	PUV	1	Surgical	50		Yes
14	17	PUV	1	Surgical	50		Yes
15	NA	Live	1	Surgical	200		NA
16	77	RPOC	1	Expectant	NA		NA
17	45	Live	1	Surgical	100		NA
18	79	RPOC	2	Surgical	1100		NA
19	49	PUV	1	Expectant	NA		NA
20	45	Live	1	Surgical	1000		NA
21	52	Live	1	Surgical	50		NA
22	60	RPOC	3	Hysterectomy	500		NA
23	82	Miscarriage	1	Surgical	50		

GA = gestational age. NA = not available. PUV = pregnancy of uncertain viability. RPOC = retained products of conception.
